# S(n)orting Out the Problem: Fever of Unknown Origin and Unexplained Abnormal Transaminases in a Young Male

**DOI:** 10.7759/cureus.1543

**Published:** 2017-08-05

**Authors:** Cyriac A Philips, Mathews J Chooracken, Pushpa Mahadevan, Philip Augustine

**Affiliations:** 1 Gastroenterology, Cochin Gastroenterology Group, Kochi, IND; 2 Department of Gastroenterology and Hepatology, Chazhikatu Hospital, Thodupuzha, IND; 3 Pathology, VPS Lakeshore Hospital, Kochi, IND

**Keywords:** cocaine, hepatitis, hepatotoxicity, dili, mdma

## Abstract

Cocaine-induced toxicity is seldom described in humans and interesting interactions with other ‘recreational’ drugs are rarely presented, mostly in animal models. We present an interesting and rare case of a young male with a fever of unknown origin and elevated transaminases in whom the cause was found to be secondary to drug-induced liver injury, confirmed on liver histopathology, related to cocaine abuse.

## Introduction

Cocaine affects multiple organ systems, including the heart, muscle, kidney, brain, gastrointestinal tract, and the liver, and has been studied extensively in mice and reported in a few human clinical cases [1-2]. The liver injury ranges from a minimal elevation of liver enzymes to severe hepatic failure, and when associated with rhabdomyolysis, has very poor prognosis [3]. We present a case of cocaine-induced liver injury in a young male presenting with fever of unknown origin and unexplained transaminases in whom a retrospective history evaluation led to a diagnosis of cocaine-induced hepatotoxicity. We also present distinct liver histopathology characteristics and drug interactions of cocaine with 3,4-methylenedioxymethamphetamine, a feature seldom described in humans. 

## Case presentation

The patient is a 26-year-old male, a non-alcoholic, non-smoker without known comorbidities, who was referred for evaluation of a fever of unknown origin of three weeks duration, abnormal liver function tests of two weeks duration, loss of appetite, and weight loss without associated jaundice, pruritus, alteration in behavior, or bleeding diathesis. Clinical evaluation revealed emaciation without pallor, lymphadenopathy, skin, or nail changes but with small mucosal ulcers of the nasal passages, a soft and non-tender hepatomegaly without splenomegaly, ascites, and a normal cardiovascular and respiratory system examination. Blood investigations revealed hemoglobin 13.8 g/L (normal 12 to 14), total white cell count 10,200 cells per mm3 (4,000 – 10,000), platelet count 2.5 x 109/L (1.5 – 4.5), total bilirubin 1.2 mg/dL (<1.5), aspartate transaminase 338 IU/L (up to 40), alanine transaminase 946 IU/L (up to 42), alkaline phosphatase 164 IU/L (up to 160), gamma glutamyl transpeptidase 138 IU/L (up to 180) and normal renal function and serum electrolytes. Evaluation for tuberculosis, malaria, leptospirosis, Rickettsia, and Brucella was noncontributory. Serum markers for hepatotropic viruses (A, B, C, and E) and human immunodeficiency virus type - 1 and 2 were non-reactive; atypical viral infections including cytomegalovirus, Epstein-Barr virus, parvovirus B and herpes group of viruses were negative. Chest and abdominal imaging followed by bone marrow aspiration, biopsy, and culture studies were within normal limits.

**Figure 1:**
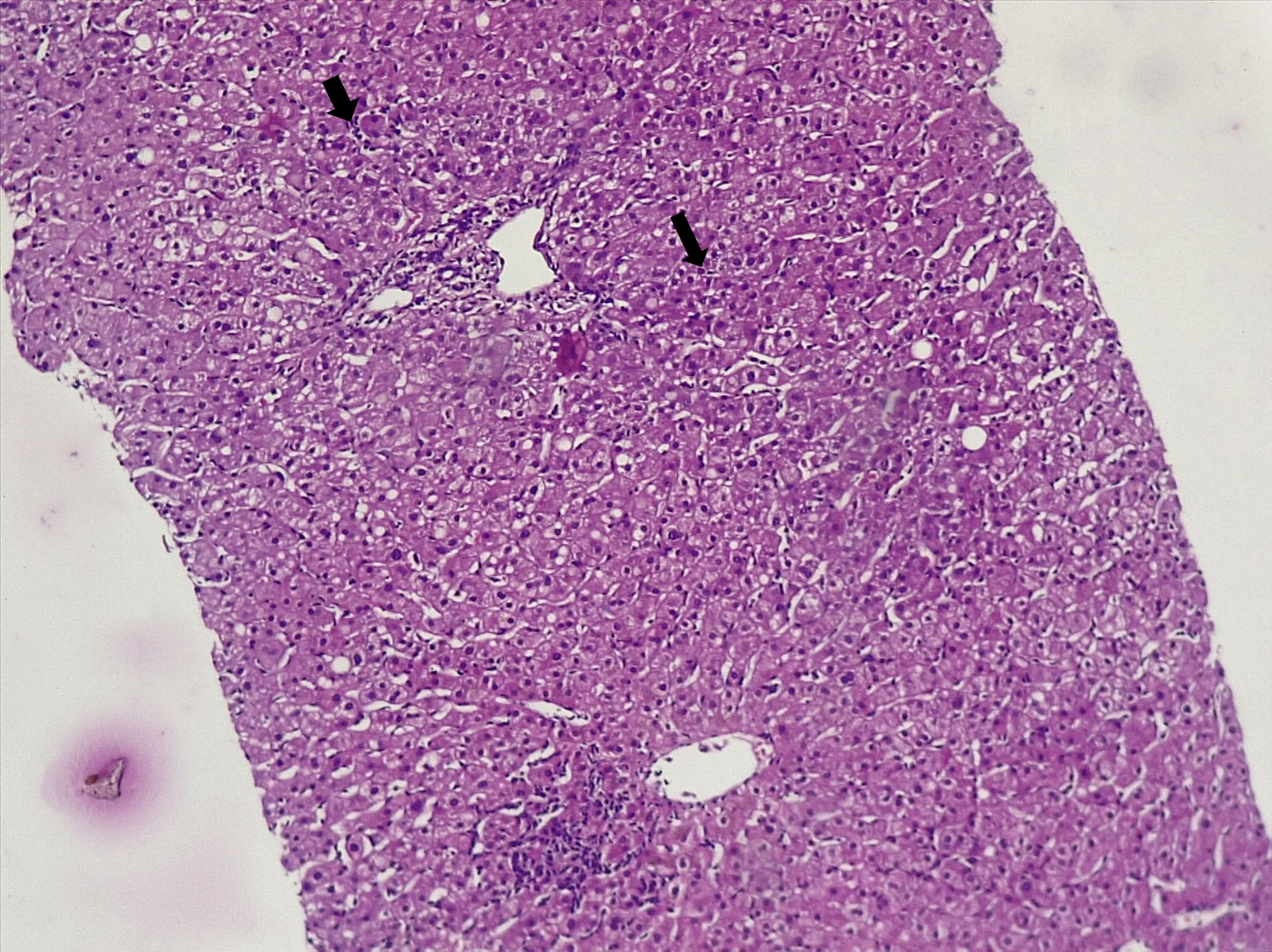
Histopathology of the liver showing lobular inflammation (arrows, hematoxylin and eosin stain, 10x)

**Figure 2:**
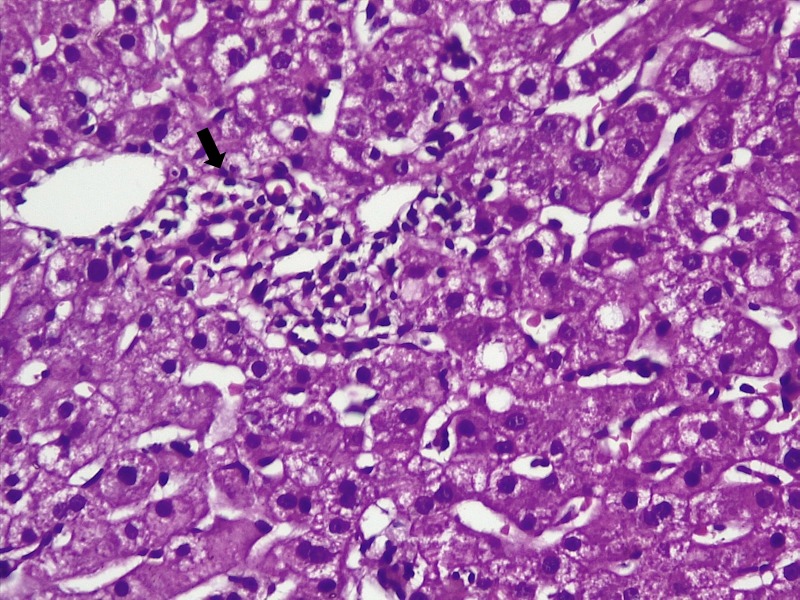
Liver histopathology showing portal-based inflammation or triaditis (arrow, hematoxylin and eosin stain, 20x)

**Figure 3:**
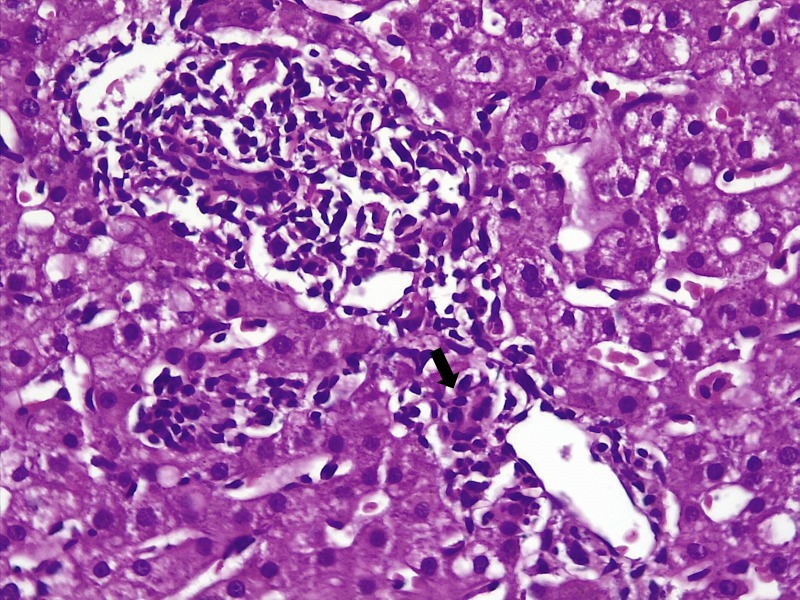
Histopathology of the liver showing central venulitis (arrow, hematoxylin and eosin stain, 20x)

**Figure 4:**
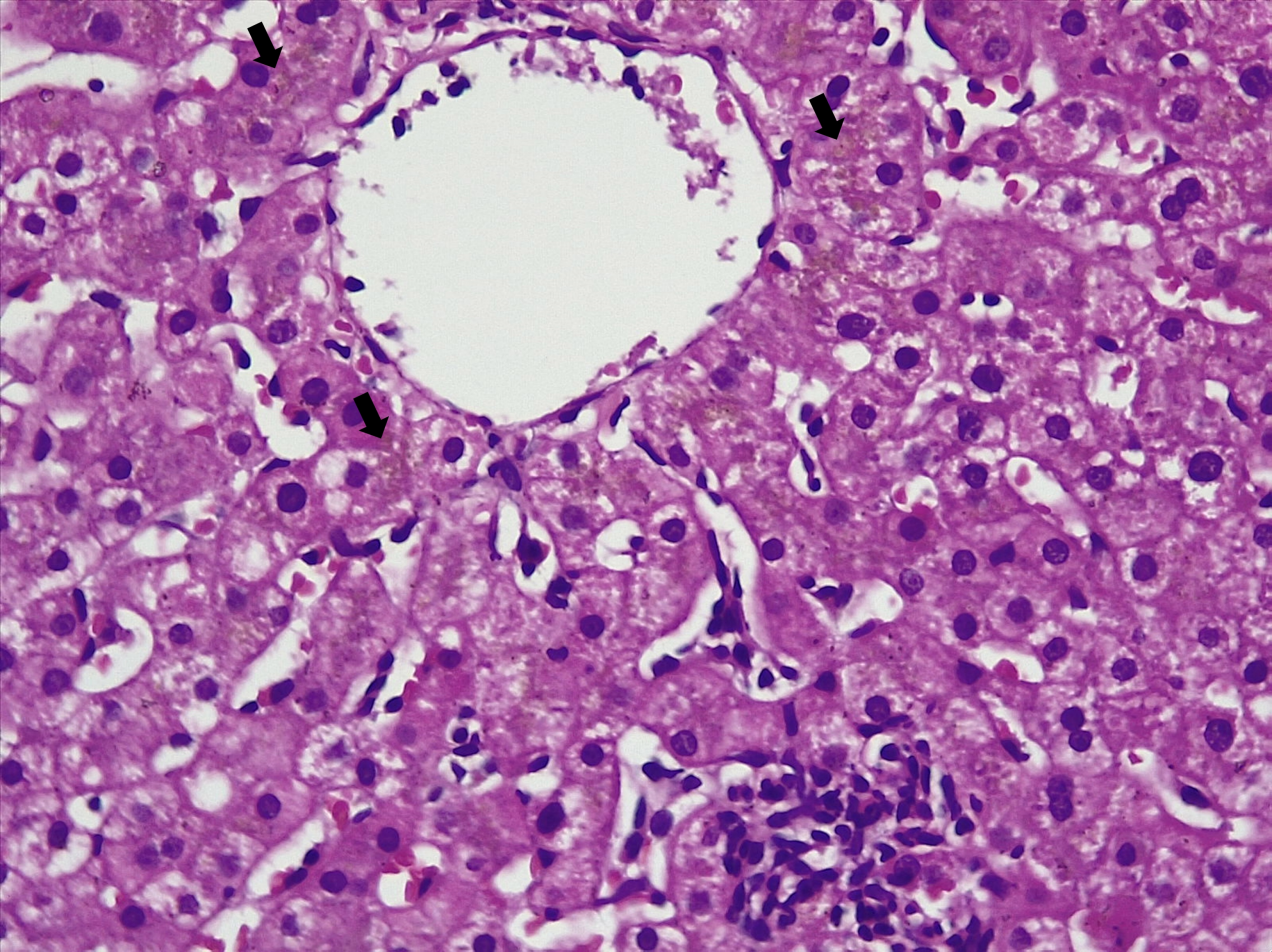
Histopathology of the liver showing extensive perivenular cholestasis (arrows, hematoxylin and eosin stain, 20x)

During the hospital stay, the fever subsided with persistence of abnormal transaminases. On repeat history, taken for complementary medication use and travel, the patient surprisingly confessed to snorting large amounts of cocaine and occasional 3,4-methylenedioxymethamphetamine (MDMA, Ecstasy) intake during a trekking trip with a group of ‘naturalists’ for five days, three weeks prior to the current presentation. A liver biopsy was performed thereafter. Liver histopathology revealed lobular inflammation (Figure 1, arrows), portal triaditis (Figure 2, arrow), central venulitis (Figure 3, arrow), and perivenular cholestasis (Figure 4, arrows) with acidophilic bodies surrounded by inflammatory cells without granulomas, atypical cells, or viral inclusions, suggestive of drug-induced liver injury.

On subsequent follow-up of over 12 weeks and in due course of time, the liver function tests improved substantially with the withdrawal of offending agents. The serial liver function test on follow-up is shown in Table 1.

**Table 1:** Serial Liver Function Tests on Follow-up

Timeline	Total bilirubin (mg/dl)	Direct bilirubin (mg/dl)	Aspartate transaminase (IU/L)	Alanine transaminase (IU/L)	Alkaline phosphatase (IU/L)	Gamma glutamyl transpeptidase (IU/L)
Baseline	1.2	0.3	338	946	164	138
Week One	1.0	0.4	317	739	156	129
Week Two	1.2	0.4	283	438	163	131
Week Four	0.9	0.2	124	202	98	98
Week Six	1.3	0.4	54	107	92	88
Week Nine	1.2	0.3	32	43	80	90
Week Twelve	1.0	0.2	38	48	81	92

## Discussion

Cocaine use can lead to a multitude of adverse effects, including coronary artery spasm, arrhythmias, myocardial infarction, cerebrovascular infarction and bleeds, seizures, intestinal ischemia, renal infarction, rhabdomyolysis, and acute liver injury. Liver injury customarily arises within hours to a few days after an acute overdose. Initially, in severe overdose, serum aminotransferase and lactate dehydrogenase levels increase markedly with minimal increase in alkaline, leading to rapid disseminated intravascular coagulation. Hyperbilirubinemia is usually seen after the third day [4]. In self-limited cases, recovery is rapid, and serum aminotransferase levels usually return to normal within one to two weeks. In our patient, the improvement took longer, possibly due to concomitant multi-drug abuse. Cocaine-induced hepatotoxicity (documented in humans, well studied in rodents) ranges from mildly elevated transaminases to severe hepatic failure associated with rhabdomyolysis and is a cause for drug fever [5]. In experimental animal models, modulation of P450 activity by inducers, inhibitors, or alcohol changes the relative toxicity and pattern of injury from cocaine. In humans, it is less clear whether the hepatic injury is mediated by a toxic metabolite of cocaine as opposed to the direct effects of hyperthermia, anoxia, or hepatic ischemia [6]. Liver histology usually shows centrilobular or zone three necrosis, portal triaditis, and microvesicular steatosis that resemble ischemic hepatitis, or hyperthermia-induced liver injury [7]. Zonality and components of injury vary according to activity/modulation of cytochrome P450 enzymes [8-9], as was seen in our patient. The concomitant use of MDMA could have led to the absence of steatosis and more of portal-based and central venulitis with cholestatic injury, the latter seen more with MDMA [10]. Fever of unknown origin and unexplained elevated transaminases warrants an exhaustive history taking with focused dissection of drug history. 

## Conclusions

Cocaine toxicity should be considered as a cause of fever of unknown origin. Young males with unexplained transaminase elevation must be worked up for recreational drug use. Multiple drug interactions can alter the histological pattern of liver injury in susceptible patients. Recreational drug use is an important cause of anicteric liver injury.
